# Socio-economic determinants of life expectancy in Nigeria (1980 – 2011)

**DOI:** 10.1186/s13561-014-0037-z

**Published:** 2015-02-07

**Authors:** Peter I Sede, Williams Ohemeng

**Affiliations:** 1 Department of Economics and Statistics, University of Benin, Edo state, Nigeria; 2 Ghana Institute of Management and Public Administration (GIMPA), Accra, Ghana

**Keywords:** D6, H75, R5, Life expectancy, Co-integration model, Nigeria

## Abstract

Attainment of 70 years life expectancy by 2020 is one of the millennium development goals in Nigeria. This study examined the socio-economic determinants of life expectancy in Nigeria using data from 1980-2011. Judging from the endogeneity feature of the variables, A VAR and VECM frameworks were employed. Socio-economic features were proxy by secondary school enrolment, government expenditure on health, per capita income, unemployment rate and the Naira foreign exchange rate. It was found that, the conventional socio-economic variables such as per capita income, education and government expenditure on health considered to be highly effective in determining life expectancy of developing countries are not significant in the case of Nigeria. The study however suggests that, life expectancy in Nigeria could be improved if attention is given to quality of government health expenditure, unemployment and measures to halt the depreciation of the Nigerian Naira against major foreign currency.

## Background

Life expectancy is a measure of the length of life expected to be lived by an individual at birth. Improvement of Life expectancy to at least 70 years by 2020 is one of Nigeria’s health policy targets. Life expectancy is frequently utilized and analyzed in the composition of demographic data for the countries of the world, for the attainment of mortality experiences and for more reliable international comparisons [1]. Jie et al. [2] and Courtney et al. [3], noted that life expectancy has important implications for the individuals and aggregate human behavior. They noted that it has crucial effects on fertility behavior, economic growth, human capital investment, intergeneration transfers and incentives for pension benefits. Granstein and Kanganovich [4], noted from the social planner’s perspective that life expectancy has implication for public finance.

Life expectancy is very crucial to the developing worlds who are earnestly striving for achieving socio-economic progress through investing significantly in social sectors like health, education, sanitation, environmental management and sustainability, and social safety nets [5]. In Nigeria, as in other developing countries, variations in morbidity and mortality have been associated with a wide variety of measures of socio-economic status including per capita GDP, fertility rate, adult illiteracy rate, per capita calorie intake, health care expenditure, access to portable drinking water, urban inhabitants, unemployment rate and the nominal exchange rate.

**Figure 1:**
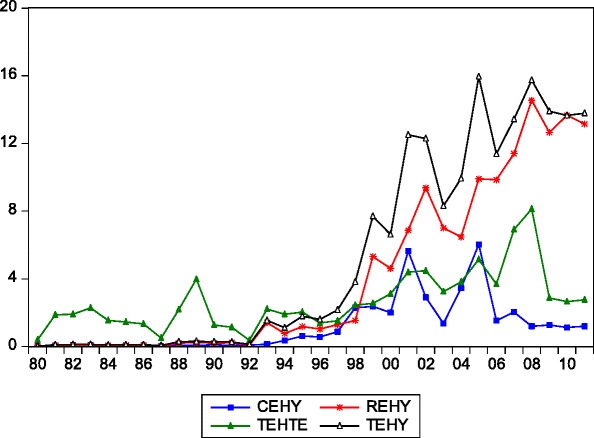
**The line graph of health expenditure indicators as percentage of GDP.** TEHY = Total expenditure on health of GDP. (% TEH of GDP); REHY = Recurrent expenditure on health of GDP. (REH as % of GDP); TEHTE = Total expenditure on health of TE. (TEH as % of TE); CEHY = Capital expenditure on health of GDP. (CEH as % of GDP).

Studies have shown that there is a significant tendency for mortality to be lower in countries with a more even distribution of income (see Wilkinson, [6]; Rodger, [7]; Le Grand [8]), but Nigeria is said to be highly non-egalitarian in income distribution. Per capita income of developing countries has improved significantly and translated into higher level of health care expenditure [5]. For instance there has been remarkable improvement in the incidence of income and non-income poverty overtime that have impacted positively on life expectancy. However, in many of the countries in Sub-Saharan Africa and Nigeria in particular, although income and health expenditure is increasing (Figure 1), life expectancy has been unsteady. An analysis of three decile averages show that between 1980 and 1989, in Nigeria, life expectancy averaged 45.8 years; 1990 and 1999, it was 45.6 years; and 2000 and 2010, it improved marginally to an average 58.6 years. Bello-Imam [9] compared the Nigerian data with the sub-region and concluded that maternal mortality rate per 100,000 live births in Nigeria averages 1,100 as against 900 Sub-Saharan African average; malaria mortality rate per 100,000 population of 156 as against 104 Sub-Saharan African average; tuberculosis mortality rate among HIV negative people per 100,000 population of 63 as against 51 Sub-Saharan African (SSA) average. Again a thirteen year average (1999-2011) data on life expectancy, under five infant mortality rate, per capita income and unemployment rate for Nigeria, Ghana, Kenya, China, India^a^ shows that Nigeria performed poorly on all these indicators (Table 1).

**Table 1:** Selected Health Indicators

**Country**	**LEXP**	**INFM**	**GDP/C**	**UNEM**
Nigeria	48.95	84.79	1,556.54	7.6
Ghana	58.72	52.61	2,121.54	4.12
Kenya	52.37	59.09	1,370.76	9.43
USA	77.79	6.44	41,723.08	6.83
China	72.83	22.6	5,925	4.23
India	65.65	50.60	2,970	3.90
Japan	81.64	3.15	30,658.33	4.51

Source: Author’s computation from Central Intelligence Agency (CIA) 2011 and International Monetary Fund 2011.

LEXP represent Life Expectancy. INFM represent Infant mortality rate. GDP/C represents Gross Domestic Product per capita Price Purchasing Parity. UNEMP represents Unemployment.

In an attempt to improve the aforementioned indicators and work towards the attainment of 70 years life expectancy by 2020, the Nigerian government has since 1980 stepped up her policy focused on the health sector through reforms and several health intervention programmes including the primary health care (PHC) intended to impact positively on life expectancy, the commercialization policy which was aimed at injecting some measure of efficiency into the public hospitals, the National Health Insurance Scheme (NHIS) initiated to mitigate the cost of access and the efficient health service delivery monitory policy (Ministry of Health, [10]) etc. However, Sede and Ohemeng [11] noted large scale inefficient utilization of available resources in most public hospitals in Nigeria. This culminates into technical and scale inefficiencies, notwithstanding the upwards trends in percentage shares of total health expenditure in GDP and total health expenditure in total government expenditure in Nigeria over time (Figure 1). The pertinent questions therefore are:

i. To what extent have health policy efforts and other socio-economic variables influenced life expectancy of Nigerians?
ii. What is the causal direction between life expectancy and some of these socio-economic variables?

These are the issues this study investigates since previous studies in this area were focused on other countries, at different times, and with different measures of socio-economic indicators suggesting that the underlying subjects may be important and should be pursued further. Nevertheless, most of the studies concentrated more on the biological, health behavioural and cultural factors. It is on the basis of these, the present study opted to investigate the effects of the socio-economic environment as constituted by per-capita income, health policy, literacy, the naira exchange rate and unemployment rates, on life expectancy of Nigerian.

The theoretical foundation of the study hinges on Grossman [12] who asserted that economic disposition of an individual is critical to affordability of health consumption. He also affirmed that the socio disposition of the individual as shown in the level of education, sense of awareness of health practices and access to health determines the health of the individual. For one thing, socio-economic variables are those factors that bother upon the social and economic conditions prevailing in the economy where the individual subsists.

Bichaku et al. [13] examined the determinants of health status (as measured by life expectancy at birth) in SSA based on the Grossman [12] theoretical model which considers the economic (the ratio of health expenditure to GDP and the per capita food availability index), social (illiteracy rate and alcohol consumption) and environmental factors (urbanization rate and carbon monoxide emission per capita index). Overall, the study showed that health policy that may focus on the provision of health services, family planning programmes and emergency aids to exclusion of other demographic issue may serve little in the schemes aimed at improving the current health status and for that matter the life expectancy at birth of the region.

## Methods

In this section, special attention is devoted to the time series component of the data series under consideration. When dealing with time series data, it is important to investigate whether the series are stationary or not because the regression of non-stationary series on another may yield spurious results. According to Engle and Grange [14], the parameter estimates from such regression may be biased and inconsistent. We applied the most widely used unit root tests for this study, that is, the Augmented Dickey-Fuller (ADF) test proposed by Dickey and Fuller [15] and Philips-Perron [16]. A concurrent test to determining the long-run relationship among variables under investigation is conducted by employing the Johansen co-integration test [17]. This is important because variables that fail to converge in the long-run may be hazardous to policy making.

Another common problem with empirical investigations is that they often ignore the feedback effect among variables in the model. In order to address this problem, vector auto-regression (VAR) is used in this study. In a VAR, each variable is regressed on its own lag and lags of other variables in the model. In this way, the procedure allows each to be affected by its own history and the history of each variable thus minimizing the problem of simultaneity [18].

The VAR contains several procedures for evaluating relationships. Two of the procedures are adopted in this study namely causality test and variance decomposition. The causality test is used to determine whether the impact of expanse in socio-economic variables on life expectancy is statistically significant. While the causality test indicates this, it may not show the relative magnitude of the impact. Therefore, the variance decomposition is used to determine the relative magnitude of such impact. More specifically, it indicates the percentage change in life expectancy that may be attributed to the effect of expansion in socio-economic variables. Such estimates are mostly useful for analyzing impacts in a multivariate system as clearly demonstrated by [19] and [20].

The study covers the period 1980-2011 (31years) which has sufficient degree of freedom to capture a considerably large proportion of the effect of socio-economic variables on life expectancy in Nigeria over time.

### Justification of the variables

Recent studies dedicated to examining possible determinants of life expectancy have considered varied variables like income, education, expenditure on health care and composite consumables, access to portable water and safe sanitation, quality energy, employment rate, residential tenure and many others. In this study variables considered to constitute socio-economic variables are: per capita income, health expenditure, literacy, and the nominal exchange rate and unemployment rate.

Income has been reported as a determinant of life expectancy in most studies. It has been established that absolute level of income measured by per capita GDP seems to impact significantly on mortality as income increases from the lowest towards the middle range of income bracket, and no further gains in life expectancy accompanies increases in income beyond certain threshold of income bracket [6]. Accordingly, Wilkinson [6] proposed that if there are diminishing health returns to increases in income, income redistribution might be pareto improvement since redistribution of income makes at least the poor better off without making those within the higher income bracket worse off. He also noted that, such income life expectancy relationship constituted a non-linear relation. However, Anand and Ravallion [21] found a significant positive linear relationship between per capita GNP and life expectancy, which is transmitted through public expenditure on health. But when poverty was introduced into their model, the relationship between per capita GNP and life expectancy became insignificant. Sen [22] reported impressive high life expectancy in the Indian state of Kerala even at low level of per capita income.

Literacy provides the individual with common social virtue of writing, reading and cultivation of health ethics which has a bearing on improving life expectancy. Education augments labour market productivity and income growth, and an educated woman has beneficial effects on child health and social well-being [5]. In analyzing 40 to 97 countries, Williamson and Boehmer [23] concluded that education impact positively on female life expectancy. There is however, contention in the literature about significant differences between mortality and life expectancy in relation to education (see Kalediene and Petrauskiene, [24]; Grabauskas and Kalediene, [25]). Rogers and Wofford [26] investigated life expectancy for 95 developing countries and came to a conclusion that literacy significantly explained the variation in life expectancy in these countries. This assertion was upheld by Gulis [27] when he employed multivariate regression analysis on 156 countries. In Nigeria, secondary school education to a very large extent is free and accessible to all children of that age in all the states of the federation. The standard of secondary educations in Nigeria is sufficient to accomplish the targets of writing, reading and access to health ethics awareness. It is the best statistic that yields the literacy level in Nigeria compared to literacy rate that might be perverted by inclusion of tertiary enrolment that might not be all encompassing.

Health policy on its part is government systematic control of important health variable, such as government expenditure on health, so as to make healthy life available and accessible to the individuals. Such policy efforts might have significant influence on life expectancy since they directly help in reducing morbidity and mortality. Cremieux et al. [28] investigating a cross province study in Canada reported that, given the various socio-economic variables in the province, lower spending is associated with a statistically significant increase in infant mortality and therefore a decrease in life expectancy. Evidence abounds in the literature concerning the positive relationship between right health policy and life expectancy. Kabir [5] reports of Costa Rica attainment of the highest life expectancy among the developing world, 74 years and 78 years in 1985 and 2002 respectively. This remarkable feat was achieved by the right health interventions, notably a primary health care programme [5]. Evidence also shows that there is positive relationship between health care inputs such as number of doctors, hospital beds, government health expenditure and health outcome (Grubaugh and Santerre, [29]; Elola et al. [30] and Novignan et al. [31]). Hitiris and Posnet [32] found a relatively small negative relationship between health expenditure and mortality rates in a cross country study.

The Naira exchange rate indirectly affects affordability of the health bills since part of the health goods and services have foreign content, whose import charges would always feed into individual final bills.

Unemployment rate would affect the affordability of hospital bills negatively. It can also affect social disposition of individual and the grade of health facilities patronized (see [6]). Based on the above reasons and availability of data these variables were adopted.

### Model specification

According to Sims [19] and Todd [20], if there is true simultaneity among a set of variables, there should not be a-priori distinction between endogenous and exogenous variables. It was on this background they developed the “vector auto-correlation” model (VAR) based on Granger causality test. The VAR model of life expectancy and the socio-economic variables in Nigeria posits that the variables are inter-related. Government health expenditure can be used to proxy government health policy. The inter-relationship of the variables is shown in the model below:

1 $$ \begin{array}{l} LLEX{P}_t = {\alpha}_{1t}\\ {}\kern5.5em +{\displaystyle \sum_{j=1}^k}{\beta}_{1j} LLEX{P}_{t-j}+{\displaystyle \sum_{j=1}^k}{\lambda}_{1j} LSECE{R}_{t-j}+{\displaystyle \sum_{j=1}^k}{\theta}_{1j}LGH{E}_{t-j}\\ {}\kern8.5em +{\displaystyle \sum_{j=1}^k}{\Omega}_{1j} LEXR{T}_{t-j}+{\displaystyle \sum_{j=1}^k}{\psi}_{1j}LUP{R}_{t-j}+{\displaystyle \sum_{j=1}^k}{\delta}_{1j}LPC{I}_{t-j}+{\mu}_1.\end{array} $$

2 $$ \begin{array}{l}\  LSECE{R}_t = {\alpha}_{2t}\\ {}\kern3.72em +{\displaystyle \sum_{j=1}^k}{\beta}_{2j} LLEX{P}_{t-j}+{\displaystyle \sum_{j=1}^k}{\lambda}_{2j} LSECE{R}_{t-j}+{\displaystyle \sum_{j=1}^k}{\theta}_{2j}LGH{E}_{t-j}\\ {}\kern3.72em +{\displaystyle \sum_{j=1}^k}{\Omega}_{2j} LEXR{T}_{t-j}+{\displaystyle \sum_{j=1}^k}{\psi}_{2j}LUP{R}_{t-j}+{\displaystyle \sum_{j=1}^k}\begin{array}{c}\hfill {\delta}_{2j}LPC{I}_{t-j}+{\mu}_{2t}.\hfill \\ {}\hfill \hfill \end{array}\end{array} $$

3 $$ \begin{array}{l}LGH{E}_t = {\alpha}_{3t}+{\displaystyle \sum_{j=1}^k}{\beta}_{3j} LLEX{P}_{t-j}+{\displaystyle \sum_{j=1}^k}{\lambda}_{3j} LSECE{R}_{t-j}+{\displaystyle \sum_{j=1}^k}{\theta}_{3j}LGH{E}_{t-j}\\ {}\kern2.88em +{\displaystyle \sum_{j=1}^k}{\Omega}_{3j} LEXR{T}_{t-j}+{\displaystyle \sum_{j=1}^k}{\psi}_{3j}LUP{R}_{t-j}+{\displaystyle \sum_{j=1}^k}\begin{array}{c}\hfill {\delta}_{3j}LPC{I}_{t-j}+{\mu}_{3t}.\hfill \\ {}\hfill \hfill \end{array}\end{array} $$

4 $$ \begin{array}{l} LEXR{T}_t = {\alpha}_{4t}\\ {}\kern3.24em +{\displaystyle \sum_{j=1}^k}{\beta}_{4j} LLEX{P}_{t-j}+{\displaystyle \sum_{j=1}^k}{\lambda}_{4j} LSECE{R}_{t-j}+{\displaystyle \sum_{j=1}^k}{\theta}_{4j}LGH{E}_{t-j}\\ {}\kern3.6em +{\displaystyle \sum_{j=1}^k}{\Omega}_{4j} LEXR{T}_{t-j}+{\displaystyle \sum_{j=1}^k}{\psi}_{4j}LUP{R}_{t-j}+{\displaystyle \sum_{j=1}^k}\begin{array}{c}\hfill {\delta}_{4j}LPC{I}_{t-j}+{\mu}_{4t}.\hfill \\ {}\hfill \hfill \end{array}\end{array} $$

5 $$ \begin{array}{l} LUPR={\alpha}_{5t}+{\displaystyle \sum_{j=1}^k}{\beta}_{5j}LEX{P}_{t-j}+{\displaystyle \sum_{j=1}^k}{\lambda}_{5j} LSECE{R}_{t-j}+{\displaystyle \sum_{j=1}^k}{\theta}_{5j}LGH{E}_{t-j}\\ {}\kern2.4em +{\displaystyle \sum_{j=1}^k}{\Omega}_{5j} LEXR{T}_{t-j}+{\displaystyle \sum_{j=1}^k}{\psi}_{5j}LUP{R}_{t-j}+{\displaystyle \sum_{j=1}^k}\begin{array}{c}\hfill {\delta}_{5j}LPC{I}_{t-j}+{\mu}_{5t}.\hfill \\ {}\hfill \hfill \end{array}\end{array} $$

6 $$ \begin{array}{l} LPCI={\alpha}_{6t}+{\displaystyle \sum_{j=1}^k}{\beta}_{6jL}LEX{P}_{t-j}+{\displaystyle \sum_{j=1}^k}{\lambda}_{6j} LSECE{R}_{t-j}+{\displaystyle \sum_{j=1}^k}{\theta}_{6j}LGH{E}_{t-j}\\ {}\kern3.12em +{\displaystyle \sum_{j=1}^k}{\Omega}_{6j} LEXR{T}_{t-j}+{\displaystyle \sum_{j=1}^k}{\psi}_{6j}LUP{R}_{t-j}+{\displaystyle \sum_{j=1}^k}\begin{array}{c}\hfill {\delta}_{6j}LPC{I}_{t-j}+{\mu}_{6t}\hfill \\ {}\hfill \hfill \end{array}\end{array} $$

Where: LLEXP_t =_ Log of Life expectancy of the economy over time.

LSECER_t_ Log of = Secondary School enrolment over time.

LGHE_t_ = Log of Government health expenditure over time.

LEXRT = Log of Exchange rate in the Nigerian economy over time.

LPCI = Log of Per-capita Income in the economy over time.

J = 1, 2, …, 6.

K = Total number of lags.

*α* = Autonomous term.

*β*_*ij*_ = Coefficients of life expectancy.

*λ*_*ij*_ = Coefficient of Secondary school enrolment.

*θ*_*ij*_ = Coefficient of Government Expenditure on Health.

Ω_*ij*_ = Coefficient for exchange rate.

*ψ*_*ij*_ = Coefficient for Unemployment.

*δ*_*ij*_ = Coefficient for per capita income.

*μ*_*it*_ = Stochastic error term (Table 2).

**Table 2:** **Data description and sources**

**Variable**	**Description**	**Sources**
LLEXP	Log of Life expectancy	World Development Indicator
LSECER	Log of Secondary school enrolment rate per population per year. It was used to proxy literacy rate as a social factor and ability to keep simple health ideals.	World Development Indicator; World Bank data 2011.
LGHE	Log of Government expenditure on health. It is used to proxy government policy on health	World Development Indicator; World Bank data 2011.
LPCI	Log of Per-capita income. It is the ratio of the country’s GDP and the population. It is used to proxy wealth as a determinant of life expectancy	World Development Indicator; World Bank data 2011.
LEXRT	Log of Exchange rate. It is used to proxy cost of living effects on life expectancy. Especially as Nigeria is an import dependent economy.	Central Bank of Nigeria (CBN) statistical bulletin Several issues.
LUNPR	Log of Unemployment.	Central Bank of Nigeria (CBN) annual report several issues, CBN statistical reports several issues [33], cited in Derived from data on UPR.
	It is another variable for welfare/economic condition of the populace.	

Source: World Bank and CBN.

Note: The log value of the data was taken, so that the coefficient estimates of the covariates can also be read as elasticity. Life expectancy figures were computed on yearly basis where a year was taken to be 365 days.

## Results

### Unit root test

In order to avoid producing spurious results that would make estimate biased and inconsistent, the time series data for all the variables in the study were tested within the period of 1980-2011 to determine their stationarity status. The results from the test based on Augumented Dickey Fuller and Philip-Perron are reported in Table 3.

**Table 3:** **Unit root test results**

	**Augumented Dickey-Fuller**				**Philip-Perron**			
	**Without Intercept**		**With Intercept**		**Without Intercept**		**With Intercept**	
	**Levels**	**1** ^**st**^ **Diff**	**Levels**	**1** ^**st**^ **Diff**	**Levels**	**1** ^**st**^ **Diff**	**Levels**	**1** ^**st**^ **Diff**
LLEXP	−2.19	−7.56**	−2.87	−7.31**	−1.90	−7.00**	−2.82	−6.85**
LGHE	−1.27	−7.04**	−1.61	−7.10**	−1.21	−7.00**	−1.53	−7.10**
LEXRT	−1.55	−4.77**	−0.94	−5.04**	−1.57	−4.77**	−1.04	−5.15**
LPCI	−0.82	−5.22**	−3.89	−5.60**	−0.08	−5.37**	−3.50	−5.75**
LUPR	−0.84	−5.73**	−2.03	−5.76**	−0.75	−5.76**	−2.00	−5.76**
LSECER	−2.72	−3.90**	−1.90	−3.95*	−2.56	−3.42*	−2.99	−5.64**

*Null hypothesis rejected at 5% **Null hypothesis rejected at 1%.

Source: Authors’ computation.

There are two test types, Augmented-Dickey Fuller and Philip-Perron. Under each test type, there are two dimensions of test results. They are, test results conducted without intercept and test results conducted with intercept. Also, under each dimension, the test was conducted at levels and at first difference. Results showed that all the variables were not stationary at levels but after first difference they were all stationary. In other words, the variables are integrated of order 1(i.e. *I*(1) series). Apart from LSECER which passed at 5% level, all others passed at 1% level of significance.

### Cointegration test

For the purposes of reasonable policy making, the relationship between macroeconomic variables in the long-run is very important. If variables have a causal relationship that allows them to move in perfect harmony in the long-run, the confidence level of the consistency of the formulated policy in their short and long run dynamics will be robust. It was against this backdrop that the co-integration test was conducted, so as to determine if there is a convergence between the long run equilibrium and the short run dynamics of the time-series data. From the test statistic of trace and maximum eigen-values, result shows that there is at least one cointegrating equation among the variables. This therefore gives the basis to reject the null hypothesis of no co-integration among the variables at 5% levels. This confirms the existence of a long run relationship between the short-run dynamics and the long run equilibrium of the model. See Table 4 below for the detailed results:

**Table 4:** **Cointegration results**

**Co-integrating Vector (LLFEXP, LGHE, LPCI, LUPR, LEXR, LSECER,)**		
**Null Hypothesis**	**Trace-Statistics**	**Maximum Eigen Statistics**
r=0	110.83*	42.37*
r = 1	68.45	25.48
r = 2	42.97	22.82
r = 3	20.15	11.83
r = 4	8.22	8.01
r = 5	0.20	0.20

*Null hypothesis rejected at 5%

Source: Authors’ computation.

### The system maximum lag length

The estimation of a VAR-model requires the explicit choice of lag-length in the equation of the model. In this study, the Akaike information criterion (AIC) was used to determine the lag length of the VAR-model. This result and that of Schwarz and Hannan-Quinn information criteria are shown in Table 5. The Akaike information criterion (AIC) is minimized for order 2. This implies that the optimal lag length of this study is order 2.

**Table 5:** **The Lag length Results of Akaike (AIC), Schwarz and Hannan-Quinn Information Criteria**

**Lags**	**Loglik**	**P(LR)**	**AIC**	**SC**	**HQ**
1	−193.15	-	11.75	13.04*	12.20*
2	−166.36	38.07	11.65*	14.02	12.49

Source: Authors’ computation.

* indicates the minimum values of the information criteria.

### Normality test

One of the requirements of regression model is that the error terms of the observations are normally distributed. The study employed the Cholesky (Lutkepohl) test to ascertain this. The results are presented below.

Results from Table 6 above show that the residuals are normally distributed as the Skewness, Kurtosis and Jarque-Bera statistics passed the chi-square test at 1%.

**Table 6:** **The Cholesky VAR normality residual test**

**Component**	**Test Criterion**	**Joint Chi-square**	**Probability**
6	Skewness	23.87**	0.00
6	Kurtosis	61.15**	0.00
6	Jarqu-Bera	81.05**	0.00

**Chi-square significant at 1%.

Source: Authors’ computation.

### Variance Inflation Factor (VIF) and tolerance colinearity test

Another test to be wary of is the collinearity of the regressors. If the regressors are correlated, the BLUE property of the model holds but, it becomes difficult to decipher the distinct impact of each of the covariates on the regressand. Collinearity becomes very worrisome when it becomes severe. If it is mild it is acceptable. Gujarati [34] and Greene [35] asserted that a VIF of more than 10 is severe while a tolerance index of zero indicates severe multicollinearity. The closer the tolerance index to 1 the milder the case. Table 7 below shows the Variance Inflation Factor and tolerance test statistics from the study data.

**Table 7:** **The Variance Inflation Factor (VIF) and Tolerance Test Statistics**

**Variable(s)**	**R**	**R** ^**2**^	**1 – R** ^**2**^ **(VIF)**	**1/1-R** ^**2**^ **(Tolerance Index)**
LLFEXP	1.0000000	-	-	-
LGHE	0.7500422	0.05626	0.9437	1.059
LEXRT	0.7551986	0.05703	0.9429	1.061
LPCI	0.670339	0,04494	0.5506	1.816
LUPR	0.743336	0.05525	0.9448	1.058
LSECER	0.866462	0.07508	0.9249	1.081

Source: Authors’ computation.

The above shows the case of mild multicolinearity among the variables as most of the tolerance indices are approximately to 1. Thus, due to mild multicolinearity the variances of regressors are inflated mildly. This is acceptable for the analysis.

### Causality test

To complement the results from the life expectancy and the stated socio-economic variables, we employed Granger causality analysis to investigate the causality relationship between life expectancy and the stated variables.

Granger [36] in his representation theorem states that, a variable X is said to granger-cause another variable say Y if past and present value of X help to predict Y. This is the traditional Granger-Causality (based on a bi-variate relationship). However, causality tests are generally sensitive to lag structure. In order to minimize this sensitivity issue, as pointed out by Gujarati [34], a VAR Granger Causality test that allows for multiple endogenous variables was considered. A lag length of 3 was chosen as per Akaike information criterion (AIC). The results are presented in Table 8.

**Table 8:** **Granger causality test results**

**Direction of causation**	**Lag length**	**F-value**	**Remark**
LPCI→ LLFEXP	3	8.69**	Reject
LLFEXP→ LPCI	3	23.31**	Reject
LGHE →LLFEXP	3	5.07**	Reject
LLFEXP → LGHE	3	0.12	Do not reject
LSECER → LLFEXP	3	1.13	Do not reject
LLFEXP → LSECER	3	16.94**	Reject
LUPR → LLFEXP	3	12.00**	Reject
LLFEXP → LUPR	3	0.68	Do not reject
LEXRT → LLFEXP	3	2.61*	Reject
LLFEXP → LEXRT	3	0.29	Do not reject

*f-statistic significant at 5% **f-statistic significant at 1%.

Source: Authors’ computation.

The results reveal that the null hypotheses that per capita income (PCI), government health expenditure (GHE) and unemployment rate (UPR) do not granger-cause life expectancy were rejected at 1% significant level. However, exchange rate (EXRT) and secondary school enrolment rate (SECER) appeared not to granger-cause life expectancy, that notwithstanding, it is striking to note that life expectancy granger-caused secondary school enrolment rate. Thus, the study found bi-directional causality between life expectancy and per capita income. Government health expenditure and life expectancy is a unidirectional relationship in favor of government expenditure granger-causing life expectancy. Life expectancy and secondary school enrolment also have a unidirectional causal relationship in favor of life expectancy causing secondary school enrolment. Similarly unemployment and life expectancy also have a unidirectional flow of relationship.

These results have some interesting implications on the Nigerian economy. To start with, the fact that PCI and LEXP are bi-causal shows that both variables reinforce each order. Thus per-capita income is necessary for enhancing life expectancy just as enhanced life expectancy is needed for improved per-capita income. Also implied from the result is that government expenditure on health is needed for enhancing life expectancy.

### Vector Auto-Regression (VAR) estimation

Since the variables in the model have been found to possess stationarity and convergence properties, the parameters of the model can as well be estimated to ascertain the relative impact of each variable on life expectancy.

### Descriptive statistics

Evidence from the Table 9 shows adjusted coefficient of determination (R^2^) of 90.5%. This shows that the explanatory variables in the model account for 90.5% systematic variations in life expectancy in Nigeria. The computed value of the F-statistics was much higher than the critical value (24.07 > 3.70) and was significant at 1% level. This therefore permits us to reject the null hypothesis that none of the explanatory variable has any significant relationship with life expectancy. The standard error estimation value indicates that the problems of inefficient parameters associated with empirical estimation of this nature are highly minimized. Also a Durbin - Watson statistics of 2.06 reveals the absence of auto-correlation. This by implication guarantees a good level of efficiency of the parameter estimates.

**Table 9:** **Parameter estimates of the Vector Autoregressive (VAR) Model**

**Variable(s)**	**Coefficients**	**t-ratio(s)**	**Other statistics**
Constant	1.88	2.93	R^2^ =0.90.5
LLFEXP(−1)	0.62	3.44**	F- Stat.=24.07**
LLFEXP(−2)	0.04	0.21	DW- Stat. = 2.06
LLGHE(−1)	−0.02	−1.52	
LLGHE(−2)	−0.004	−0.46	
LEXRT(−1)	−0.01	−0.63	
LEXRT(−2)	−0.04	1.89*	
LPCI(−1)	0.02	0.10	
LPCI(−2)	−0.08	−0.51	
LUPR(−1)	−0.02	−1.06	
LUPR(−2)	−0.03	−1.86*	
LSECER(−1)	0.04	10.29**	
LSECER(−2)	−0.10	−1.07	

**/*significant at 1% and 5% respectively.

Source: Authors’ computation.

### Secondary school enrolment and life expectancy

It is obvious from the results that the immediate past periods of secondary school enrolment significantly affect current level of life expectancy of Nigerians positively. The positive coefficient of the literacy rate is in conformity with the study by Rogers and Wofford [26] and Gulis [27] on number of developing countries. Table 9 shows that increase in one period past secondary school enrolment was more likely to increase life expectancy at birth at 1% significance level. A 1% increase in past school enrolment leads to an improvement in life expectancy at birth by approximately 15 days.

### Per capita income and life expectancy

It is striking to notice from the results that the immediate two periods of per capita income presents not only a mixed outcomes as to the direction of its impact on current life expectancy, the results are also not significant. This is in line with the findings of Anand and Ravallion [21] when poverty was introduced into their model. This means the Nigerian data might have implicitly taken into consideration the level of poverty in the country. The results are contrary to the findings of Wilkinson [6] but support the finding of Kabir [5] who examined 91 developing countries on the same subject.

### Government expenditure and life expectancy

Government expenditure on health also did not meet up with expected sign. It was negative and not significantly related with the current level of life expectancy in its immediate two past periods. This is in support of Hitiris and Posnet [34] and Kabir [5] but contradicts that of Grubaugh and Santerre, [29]; Elola et al. [30], and Novignan et al. [31]. This is expected since the percentage share of capital expenditure on health to GDP continues to exhibit downward trend compared with recurrent expenditure on health as a percentage of GDP (Figure 1). This is indicative of poor performance, in terms of government expenditure on capital inputs into healthcare. In developing countries like Nigeria where health infrastructure are largely under developed, government health expenditure are used in providing and developing health facilities and improving health systems operations (Novignan et al. [31]).

### Unemployment and exchange rates

The two immediate past period each of exchange rate and unemployment however, affect the current level of life expectancy negatively at 1% level of significant. Thus, 1% increases in two immediate past periods value of the Dollar - Naira exchange and unemployment rates reduced the current value of life expectancy by approximately 6 days respectively. Unemployment is influential factor that needs necessary attention. In Nigeria where the demographic structure is such that, the youth constitutes the greater percentage of the population, the life expectancy of the population would be enhanced if employment could be raised.

### Result of variance decomposition

The essence of the variance decomposition is to measure the proportion of error variance in one variable explained by innovations from itself and other variable. In the preceding analysis, it was established that the impact of socio-economic environment on life expectancy in Nigeria is not so significant (through the Granger causality test). The magnitude of this impact can be ascertained from variance decomposition of the VAR, which indicates the relative contribution of past period’s life expectancy to its current values as well as the contribution made by other explanatory variables to its value. The results are presented in Table 10.

**Table 10:** **Variance decomposition estimate (in percentages)**

**Period**	**LLEXP**	**LGHE**	**LEXRT**	**LPCI**	**LUPR**	**LSECER**
1	100.00	0.000	0.000	0.000	0..000	0.000
2	89.505	5.821	0.877	0.002	3.511	0.284
3	73.039	4.747	2.736	0.020	18.23	1.234
4	64.303	4.470	3.614	0.280	25.832	1.502
5	53.538	7.364	3,849	0.340	33.667	1.246
6	45.603	12.387	3.996	0.290	36.678	1.042
7	41.658	16.958	3.984	0.260	36.243	0.908
8	39.646	20.654	4.077	0.230	34.569	0.821
9	38.913	22.985	4.310	0.220	32.792	0.782
10	38.886	24.084	4.718	0.210	31.336	0.770

Source: Authors’ computation.

The variance decomposition for life expectancy alone is shown because it forms the major thrust of the study. The results which are in three lags show that apart from the share of 38% from immediate past period of itself, the largest share of the change in life expectancy comes from unemployment rate with 31%. This is followed by government expenditure on health with 24%. The next is exchange rate with a change of 4.7%. This is followed by secondary school enrolment with 0.77% and lastly by per capita income with a value of 0.21%.

Following Johansen and Juselius [37] and Johansen [17], a vector of endogenous variables, *x*, that are integrated of order 1, is analysed using the vector error correction representation (VECM). All the variables included in the model are treated as endogenous. The choice of the optimal lag lengths of the variables was determined by the multivariate forms of the Akaike Information Criterion (AIC) and the Schwartz Criterion (SC). The results from the VECM are summarized in Table 11.

**Table 11:** **The VECM results**

**Variable(s)**	**Coefficient(s)**	**t-Values**	**Standard error**	**Other statistics**
Constant	0.0056	0.8680	0.0065	F=9.9262
LLEXP(−1)	−0.0236	−0.2636	0.0895	DW=1.942
LLEXP(−2)	−0.027	−0.3562	0.0761	$\overset{\_\_}{R^2}$=0.8056
LGHE(−1)	0.0067	1.4998	0.0045	
LGHE(−2)	0.0095	2.2618*	0.0042	
LEXR(−1)	−0.0457	−3.6996*	0.0124	
LEXR(−2)	0.0460	0.4792	0.0960	
LPCI(−1)	0.0224	0.2748	0.0817	
LPCI(−2)	0.0244	0.3572	0.0683	
LSECER(−1)	0.0404	0.7594	0.0532	
LSECER(−2)	0.0367	0.6349	0.0578	
LUPR(−1)	−0.0447	−3.7563*	0.0119	
LUPR(−2)	−0.0423	−3.3848*	0.0125	
ECM	−0.4466	−6.1340*	0.0720	

*significant at 5%.

Source: Authors’ computation.

It is clear from the results that apart from the previous two periods log values of unemployment rate, the previous two period exchange rates and the previous period government expenditure on health, the conventional socio-economic variables considered to be highly effective in determining life expectancy of developing countries are not significant in the case of Nigeria. The study establishes that the improvements in terms of per capita income, educational enrolments and to some extent, government total expenditure on health may not translate into higher life expectancy. This is consistent with Kabir [5] who also confirmed that over the last ten years many of these developing countries have witnessed gains in these areas but demonstrated decrease in life expectancy.

The Nigeria situation may be explained by the high inequality among the population. Several arguments have been suggested as to why income inequality could be harmful and lower the efficiency of economic growth in reducing poverty among SSA, Nigeria inclusive. Therefore, gains in per capita income may not necessarily translate into improvements in life expectancy in the Nigeria context considering the highly non-egalitarian nature of the country.

## Discussion and policy recommendation

This study quantified the lagged effect of socio-economic factors on increased life expectancy over the 31 years of the study period in Nigeria. The five selected factors, per capita GDP, secondary school enrolment, public health care expenditure, unemployment rate and the nominal exchange rates together accounted for 90% gains in life expectancy, given a lag period of up to three years. The focal outcome of the analyses is that the traditional socio-economic variables considered to be highly prominent in determining life expectancy of developing countries were not significant in the context of Nigeria. There is no assurance that improvements in per capita income, secondary school enrolments, and to some degree, public expenditure on health may exert positive persistent effects on the life expectancy of Nigerians. However, the study suggests that life expectancy could be improved if attention is given to quality government health capital expenditure particularly expenditure on medical infrastructure, equipment and other health deliverables, as against the escalated recurrent expenditure witnessed in the health sector. Unemployment, particular youth unemployment is also significant factor that needs special attention. Unemployment in Nigeria is concentrated in the younger segment of the population (15 to 30 years old). Unemployment adversely affects the proportion of the total disposable income received by the low income households and tends to generates economic conditions that negatively affect life expectancy. This cannot be doubted judging from the negative effects on standard of living. Finally, the rate at which the Nigerian Naira depreciated to the U.S. Dollar adversely affects the life expectancy of the population. Nigeria like most developing countries is import dependent for pharmaceutical products and medical equipment. A depreciation of the local currency translated into higher cost of health services and health products which invariable cut out majority of the population which falls below the poverty line from assessing medical care. The depreciation of the Naira against the major currencies resulted in high cost of living that motivated many away from accessing orthodox medical cares.

The analysis above has serious policy implications particularly for health sector and life expectancy in Nigeria.

The outcome suggests that increased in the share of income had no influenced on the life expectancy of Nigerians. Overall, there is the tendency for mortality to fall most rapidly among countries with a more egalitarian income distribution than when income is skewed towards a very few. Therefore, for Nigeria to experience the potential benefits of improved per capita income, redistribution of the income should be the policy direction.

There is a need to improve on health policy in the area of provision of funds, efficient utilization of available resources and efficient investment in core medical equipment and service deliverables. Government in developing countries has responsibility to improve upon the provisions of goods and services including the provision of health care infrastructure, training of health personnel, immunization and other preventive health care measures.

Employment policy should be vigorously pursued. Enabling environment in terms of stable macroeconomic as well as watertight security conducive for business to triumph should be the ultimate preoccupation of the managers of the economy. If unemployment is likely to adversely affect life expectancy, a higher proportion of unemployed people would tend to increase the dependency ratio, widen income distribution and adversely affect the affordability of the unemployed to properly access medical care.

Economic diversification of production in Nigeria is a good policy direction to pay attention to. As the Naira exchange rate is determined by the free forces of the market, diverting from a mono-cultural economy (oil dependent) to a diversified one will create diverse sources of export earnings which will enhance foreign exchange reserve for the country. This inevitable reduces the menace of high exchange rate of the Naira that creates high cost of living and impacts negatively on the health of the country.

### Limitations

The study is limited in the sense that the variables used to proxy for the determinants of life expectancy may not be exhaustive; other bio-medical and environmental variables may be highly correlated with life expectancy. Nevertheless, this study is not suffering from omission biased.

## Conclusion

In spite of several economic policy efforts made by governments towards improving on life expectancy, the gains can be described as discouraging. This study therefore sought to investigate the socio-economic determinants of life expectancy in Nigeria, employing data from 1980 to 2011. The study specified a vector auto-regression VAR model taking into consideration, the endogeneity features of the economic variables specified to affect life expectancy. Five known variables relevant to Nigeria situation have been used as regressors to examine their significance in determining life expectancy. Having affirmed the robustness of the relevant statistical features of the model through the relevant diagnostic tests; the model had a good fit at 1% level; there is absence of autocorrelation and multicollinearity. The overall results showed that the socio-economic environment in Nigeria as constituted by government health expenditure, secondary school enrolment, and per-capita income have not exerted significantly on life expectancy in Nigeria. However, unemployment and nominal exchange rates effects on life expectancy have been found very relevant in the study. Thus, contrary to the previous studies, the results suggest that most of the traditional variables considered to be influential of life expectancy turned out to be insignificant. It is therefore recommended that relevant policy instruments be put in place to enhance life expectancy through the creation of favourable socio-economic environment. This can be achieved by effective manipulation of the relevant policy instruments such as redistribution of income, employment drive, and diversification of the economy away from oil dependent. These are necessary and highly important in actualizing the 70 year Life expectancy objective of Nigeria.

## Endnote

^a^Nigeria is compared to Ghana and Kenya because they are all in SSA; then, to China and India because they have similar population size.
